# Single-walled carbon nanotubes increase pandemic influenza A H1N1 virus infectivity of lung epithelial cells

**DOI:** 10.1186/s12989-014-0066-0

**Published:** 2014-12-14

**Authors:** Pallab Sanpui, Xiao Zheng, Julia C Loeb, Joseph H Bisesi Jr, Iftheker A Khan, A R M Nabiul Afrooz, Keira Liu, Appala Raju Badireddy, Mark R Wiesner, P Lee Ferguson, Navid B Saleh, John A Lednicky, Tara Sabo-Attwood

**Affiliations:** Department of Environmental and Global Health, Center for Environmental and Human Toxicology and Emerging Pathogens Institute, University of Florida, 2187 Mowry Road, Box 110885, Gainesville, FL 32611 USA; Department of Civil, Architectural and Environmental Engineering, University of Texas at Austin, 301 E. Dean Keeton Street, Austin, TX 78712 USA; Department of Civil and Environmental Engineering, Nicholas School of the Environment, and Center for the Environmental Implications of NanoTechnology, Duke University, 121 Hudson Hall, Box 90287, Durham, NC 27708 USA

**Keywords:** Single-walled carbon nanotubes, Influenza virus, H1N1, Lung epithelial cells, Infectivity, Cytokines, Interferon Induced Proteins with Tetratricopeptide repeats (IFIT)

## Abstract

**Background:**

Airborne exposure to nanomaterials from unintended occupational or environmental exposures or as a consequence of product use may lead to adverse health effects. Numerous studies have focused on single-walled carbon nanotubes (SWCNTs) and their ability to cause pulmonary injury related to fibrosis, and cancer; however few studies have addressed their impact on infectious agents, particularly viruses that are known for causing severe disease. Here we have demonstrated the ability of pristine SWCNTs of diverse electronic structure to increase the susceptibility of small airway epithelial cells (SAEC) to pandemic influenza A H1N1 infection and discerned potential mechanisms of action driving this response.

**Methods:**

Small airway epithelial cells (SAEC) were exposed to three types of SWCNTs with varying electronic structure (SG65, SG76, CG200) followed by infection with A/Mexico/4108/2009 (pH1N1). Cells were then assayed for viral infectivity by immunofluorescence and viral titers. We quantified mRNA and protein levels of targets involved in inflammation and anti-viral activity (INFβ1, IL-8, RANTES/CCL5, IFIT2, IFIT3, ST3GAL4, ST6GAL1, IL-10), localized sialic acid receptors, and assessed mitochondrial function. Hyperspectral imaging analysis was performed to map the SWCNTs and virus particles in fixed SAEC preparations. We additionally performed characterization analysis to monitor SWCNT aggregate size and structure under biological conditions using dynamic light scattering (DLS), static light scattering (SLS).

**Results:**

Based on data from viral titer and immunofluorescence assays, we report that pre-treatment of SAEC with SWCNTs significantly enhances viral infectivity that is not dependent on SWCNT electronic structure and aggregate size within the range of 106 nm – 243 nm. We further provide evidence to support that this noted effect on infectivity is not likely due to direct interaction of the virus and nanoparticles, but rather a combination of suppression of pro-inflammatory (RANTES) and anti-viral (IFIT2, IFIT3) gene/protein expression, impaired mitochondrial function and modulation of viral receptors by SWCNTs.

**Conclusions:**

Results of this work reveal the potential for SWCNTs to increase susceptibility to viral infections as a mechanism of adverse effect. These data highlight the importance of investigating the ability of carbon-nanomaterials to modulate the immune system, including impacts on anti-viral mechanisms in lung cells, thereby increasing susceptibility to infectious agents.

**Electronic supplementary material:**

The online version of this article (doi:10.1186/s12989-014-0066-0) contains supplementary material, which is available to authorized users.

## Background

SWCNTs are emerging as one of the most commercially important and technologically-relevant nanomaterials in research and consumer industries. There are numerous types of SWCNTs [1], each with unique properties that allow for molecular level manipulation and thus have enabled them to be used in a variety of industrial and consumer products [2,3] and are being intensely investigated for their use in diverse biomedical applications [4-8]. While SWCNTs are well suited for many of these applications, there is emerging concern regarding the potential for adverse health effects associated with unintended occupational or environmental exposures or intended product use such as application of biomedical or personal care products. Data regarding the potential for exposure to SWCNTs and related health risks is sparse at best. Two recent reviews anticipate that the greatest risk of exposure to SWCNTs will likely occur from product release during manufacturing, subsequent processing and product wear and tear (tires, recycling, textiles) [9,10]. With increasing manufacture and use of SWCNTs, it is imperative to thoroughly investigate nanomaterial biotoxicity in order to better evaluate associated health implications [11].

There has been remarkable interest in inhalation as a route of exposure as several *in vivo* studies report that SWCNTs can induce adverse pulmonary effects [11-13] such as subchronic tissue damage, fibrogenesis, granulomatous changes, impaired clearance, robust inflammation, airway hyper-reactivity and airflow obstruction, and cardiopulmonary effects [14]. The cellular and molecular mechanisms that contribute to these outcomes include oxidative stress, modulation of inflammatory mediators (cytokines, chemokines), genotoxicity, altered expression of stress genes, mitotic disruption, changes in biotransformation enzymes, phospholipid peroxidation, epithelial mesenchymal transition, and altered arterial baroreflex function [15-20]. The majority of these data originate from studies designed to assess the toxicity of carbon nanomaterial exposures in isolation of other imposed stressors.

It is well recognized that heightened and, in some cases, distinct biological responses can occur with co-exposure to multiple inhaled agents as is the case for synergistic free radical generation by diesel exhaust and bacterial lipopolysaccharide (LPS) [21]. Although reports are controversial, certain viruses may also act as disease co-factors with toxicants - as is postulated for SV40 and asbestos in mesotheliomas [22,23]. Only a few studies have investigated sequential exposure of nanoparticles and pathogens. These reports collectively show that co-exposure with bacteria leads to enhanced pulmonary inflammation and fibrosis and decreased pathogen clearance highlighting the potential impacts of combined exposures [24,25]. More recently, carbon nanotubes have been shown to exacerbate ovalbumin- induced airway remodeling and allergic asthmatic responses in mice [6,7,26-28].

While there are intense ongoing research efforts focused on using nanoparticles for viral detection and vaccine development [3,29], we are unaware of studies performed to date that investigate the toxicological impact of pristine SWCNTs on viral infectivity. Historical evidence highlights the causal relationship between inhaled particulates and associated lung diseases including fibrosis, cancers and exacerbation of asthma and bronchitis, conditions that may also render individuals susceptible to the pathogenicity of infectious agents, chiefly bacteria and viruses [30]. Conversely, these biologic agents may be capable of modulating the pulmonary response to inhaled particles at the nanometer scale. This can have immense consequences as infectious agents, such as influenza A, are notorious for causing global pandemics that carry heavy mortality burdens. As realistic exposure scenarios involve multiple agents, triggering of conserved mechanisms may lead to detrimental responses that contribute to more severe, and in some cases unexpected health outcomes. This underscores the critical need to understand how nanoparticles influence cell behavior, alone and in combination with familiar pathogens, acknowledging that many of these changes could have a significant impact on cell/organ function *in vivo*. Studies that define the immune defense response to nanoparticles and their modulation of anticipated pathogenic outcomes are critically lacking and have implications for long-term health effects.

Herein, we have focused our efforts on pristine non-surface functionalized SWCNTs, allotropes of carbon that are defined by the length and chiral angle of the tube roll-up vector [31]. We will use the term ‘electronic structure’ to describe the combined properties of diameter and chiral wrapping angles. We investigated the impact of three distinct types of SWCNTs that differ in their electronic structure on the infectivity of lung epithelial cells to pandemic influenza A H1N1 virus (IAV). The hypothesis that SWCNT electronic structure could affect viral infectivity is based on the assumption that the diverse surface energies associated with conductive versus semi-conductive SWCNTs could influence binding of these nanoparticles to biomolecules such as proteins or viruses. In fact, our research team has previously shown that chirality does influence both fractal structure [32] and aggregation [33] of SWCNTs in biologically relevant conditions. It is also reported that peptide binding is highly dependent on the diameter variation of the SNCNTs, which is one of the two parameters that control their electronic structure.

Results of this work reveal that SWCNTs can enhance viral infectivity of lung cells independent of electronic structure. We also provide evidence that SWCNTs inhibit IAV-induced expression of anti-viral molecules IFIT2 and IFIT3 and the cytokine RANTES, increase viral attachment receptors, and impair mitochondrial function. Taken together, these studies highlight the potential for SWCNTs to influence pathogenic infections in the lung by altering the normal anti-viral immune response of epithelial cells.

## Results and discussion

### SWCNT characterization under biological conditions

**Figure 1:**
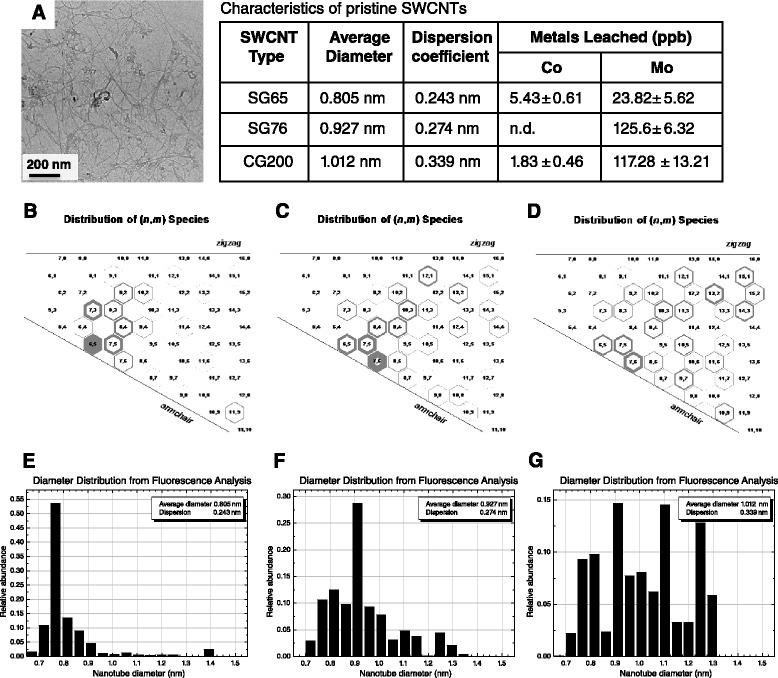
**Characterization of SWCNTs suspended in pluronic 68.** Representative TEM images of **(A)** SG65 SWCNTs, scale bar = 200 nm. Chirality distribution maps for **(B)** SG65, **(C)** SG76 and **(D)** CG200 SWCNTs. Diameter distribution measurements using near infrared fluorescence for **(E)** SG65, **(F)** SG76 and **(G)** CG200 SWCNTs.

For this work we employed a series of pristine, non-functionalized SWCNTs produced by the vapor deposition process using molybdenum/cobalt catalysts [34] and previously characterized and utilized by our group [33-38]. Although each select type of SWCNT is similar in appearance (representative TEM of SG65 SWCNTs, Figure 1A) they are each distinctly defined by their unique range of electronic structures and thus chiral wrapping vectors (Figure 1B-D). SG65 and SG76 are mixtures of predominantly semi-conducting SWCNTs that have average diameters of 0.805 nm +/− 0.243 and 0.927 +/− 0.274, respectively as determined by near-infrared fluorescence (NIRF) (Figure 1E-F) whereas CG200 are composed of primarily conductive SWCNTs with an average diameter of 1.012 +/− 0.339 (Figure 1G). All three types of SWCNTs contain < 5% (w/w) metal catalyst (approximately 2:1 molybdenum:cobalt).

**Figure 2:**
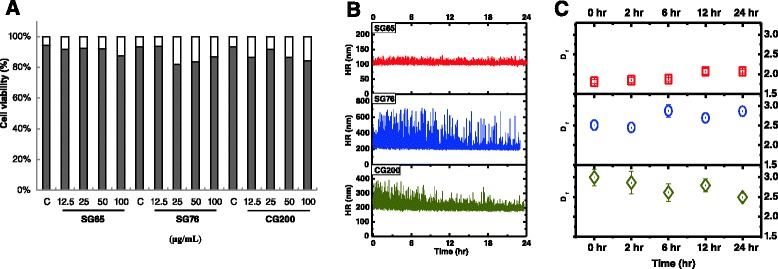
**Pluronic F68 suspended SWCNTs are not cytotoxic to small airway epithelial cells.** Cells were exposed to SG65, SG76 or CG200 SWCNTs at doses ranging from 12.5 μg/mL – 100 μg/mL. **(A)** Cytotoxicity was determined after 24 h of exposure using a trypan blue exclusion assay. Data is presented as the mean % cell viability for each treatment and represents 3 experiments combined. No statistical differences in cytotoxicity were observed for any of the does tested as determined by ANOVA (*P* < 0.05). Live cells are represented by gray bars and dead cells by white bars. Characterization of SWCNTs in cell media conditions. Each of the SWCNT stocks were incubated with cell media used for exposures and **(B)** Size distribution was measured for 24 h using dynamic light scattering (DLS). **(C)** Fractal dimension of the 3 types of SWCNTs were determined in cell culture conditions for 24 h using static light scattering (SLS).

We have characterized these particles as-prepared SWCNT mixtures for purity and diameter/chirality using several methods, including TEM, SEM, EDS, Raman spectrometry, asymmetric flow field flow fractionation, and NIRF spectroscopy published previously [38]. Further characterization of the SWCNTs was conducted under our cell culture conditions and included measurements of hydrodynamic radius (HR), fractal dimension and metal leaching by dynamic light scattering (DLS), static light scattering (SLS), and inductively coupled plasma-mass spectrometry (ICP-MS), respectively. In these characterization studies, we employed doses of SWCNTs which were shown to be non-cytotoxic to small airway epithelial cells (SAEC) (Figure 2A). Data from DLS analysis demonstrated time-dependent aggregation characteristics of the SWCNTs where SG65 nanotubes showed a stable average HR of 106 ± 5 nm for the entire 24 h exposure period (Figure 2B). The other two types of SWCNTs, SG76 and CG200, showed higher polydispersity, more instability within the first 6 h and larger sizes compared to the SG65 type, presenting with average HR of 243 ± 64 and 207 ± 26 nm, respectively. Similar aggregation propensity for larger diameter SWCNTs (both SG76 and CG200) was observed in a previous study [33]. Using SLS, Figure 2C illustrates the evolution of aggregate structure of the nanomaterials over the 24 h exposure time frame. With this technique we were able to determine fractal dimension (D_f_) which is a measure of aggregate structure. These data showed that SG65 SWCNTs formed fractal aggregates with initial D_f_ of 1.81 ± 0.05, increasing to 2.07 ± 0.05 after 24 h. D_f_ of SG76 varied from 2.51 ± 0.12 to 2.86 ± 0.12, while CG200 SWCNTs showed D_f_ variation between 2.99 ± 0.20 and 2.49 ± 0.10. Overall denser packing for SG76 and CG200 SWCNTs for the entire 24 h duration is predicted, compared to relatively loosely bound less dense fractal structures for SG65 SWCNTs. It is likely that the SG76 and CG200 samples had higher SWCNT-SWCNT interaction due to the presence of larger diameter tubes, hence higher van der Waals interaction leading to a more compact aggregate (higher D_f_) as shown in one of our recent works [32].

We also performed trace metal analysis on cell culture media incubated with each type of SWCNT for 24 h as a simulated measure of leachability and thus, bioavailability. All three types of SWCNTs tested leached higher levels of Mo into the media compared to Co. Greater levels of Mo were observed in media incubated with SG76 (125.62 ppb) and CG200 (117.28 ppb) compared to SG65 (23.81 ppb) SWCNTs. For Co, the levels for SG65 SWCNTs were 5.430 ppb followed by CG200 (1.82 ppb) and SG76 (non-detectable). While the levels of Co and Mo are quite low and not likely to be a major driver of the cellular effects described below, it can not be entirely ruled out that these metals do not contribute to surface reactivity of SWCNTs as previously suggested [39].

### Viral infectivity of SAEC

**Figure 3:**
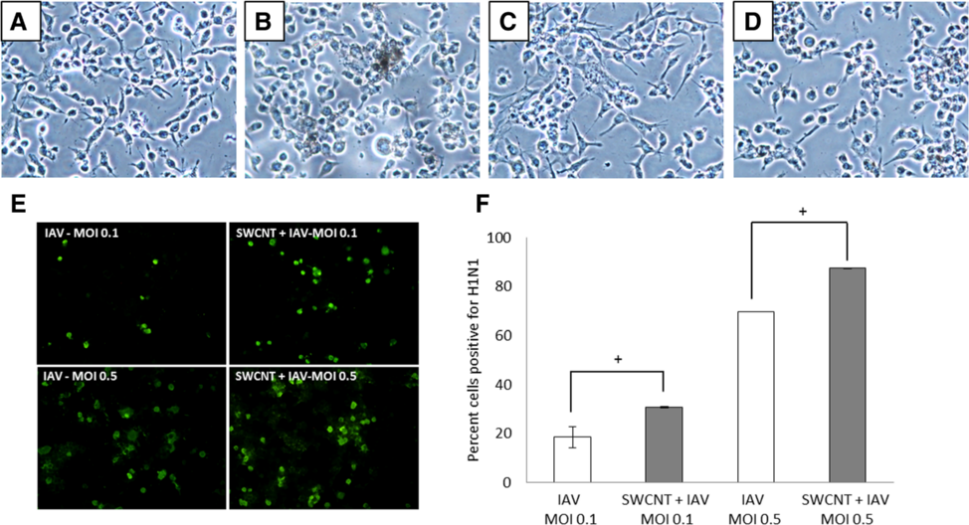
**Altered morphology and increased infectivity of SAEC infected with IAV (low dose: MOI-0.1 and high dose: MOI-0.5) with or without pre-treatment of 50 μg/ml SG65 SWCNTs for 24 h.** The panels above **(A-D)** are phase contrast images (200X) of SAEC cells exposed to **(A)** control pluronic, **(B)** SWCNTs, **(C)** IAV and **(D)** SWCNTs + IAV showing morphological differences between the treatments. **(E)** Positive immunofluorescence staining (green) of SAEC infected with IAV with or without pre-treatment of SG65 SWCNTs for 24 h. **(F)** The percent of SAEC infected with IAV is presented as the calculated mean ± standard error of 3 separate experiments. The results show that the number of infected cells increase when pretreated with SG65 SWCNTs for 24 h as determined by ANOVA. *P < 0.05.

We hypothesized that SWCNTs would variably influence viral infectivity of lung cells that was dependent on the electronic structure of the nanotubes. To test this hypothesis, we first assessed whether SG65 SWCNTs would alter the level of infectivity of SAEC with pandemic influenza A virus A/Mexico/4108/2009 (IAV). Results of these experiments showed a dose dependent increase in cell infectivity with exposure to IAV alone as observed by immunochemistry (Figure 3E). Pre-exposure of SAEC to 50 μg/mL SG65 SWCNTs for 24 h caused a significant increase in the infectivity by IAV for both a low (0.1 MOI) and high (0.5 MOI) dose of virus. The percent of total cells infected were semi-quantified from the immunofluorescent stained slides (Figure 3F). Infectivity was also quantified by determining viral titers (Table 1) which revealed a 3.5- and 5.6-fold increase in infectivity in the presence SG65 SWCNTs for both the low and high dose of virus, respectively.

**Table 1:** **Number of virus particles after 24 h of infection in the presence or absence of SWCNT pre**-**treatment as determined by titer assay**

**Treatment**		**Titer (24 h post-infection)**	**Fold increase in titer**
SWNT (type, 50 ug/ml)	Virus (MOI)		
-	0.1-Low	5.2 × 10^5^TCID_50_/ml	3.5X
SG65	0.1-Low	1.8 × 10^6^TCID_50_/ml	
-	0.5-High	3.2 × 10^6^TCID_50_/ml	5.6X
SG65	0.5	1.8 × 10^7^TCID_50_/ml	
-	0.5	3.2 × 10^6^TCID_50_/ml	5.0X
SG65	0.5	1.6 × 10^7^TCID_50_/ml	
-	0.5	3.2 × 10^6^TCID_50_/ml	5.0X
CG200	0.5	1.6 × 10^7^TCID_50_/ml	
-	0.5	3.2 × 10^6^TCID_50_/ml	-(no change)
Carbon black	0.5	3.2 × 10^6^TCID_50_/ml	

Morphological changes in cell architecture were also noted as SAEC exposed only to SWCNTs appear more rounded (Figure 3B) compared to classic cuboidal shaped control cells, where those infected with virus are more spindle shaped with granular cytoplasm and enlarged pronounced nuclei (Figure 3C). Cells with the combined exposures of SWCNTs and IAV present with a mixture of the aforementioned morphological traits (Figure 3D). At this point it is not clear what the consequence of this observed cellular ‘roundness’ is or related cellular changes at the molecular level. Based on the dye-based cell viability assay we did not observe severely compromised cell membranes although these results do not rule out more subtle changes in membrane function caused by exposure to the SWCNTs. To examine if there were differential impacts of other types of chirally-enriched SWCNTs compared to SG65 SWCNTs on IAV infectivity, additional experiments were performed where SAEC were pre-exposed to SG76 and CG200 SWCNTs (50 μg/mL) 24 h prior to IAV infection. Results from these studies show that both types of SWCNTs increased virus infectivity comparably to SG65 SWCNTs, resulting in a 5 fold increase in infectivity in the presence of SWCNTs compared to the virus alone (high dose, MOI 5.0). The presence of the control particle, carbon black, failed to increase virus infectivity in any of the exposure scenarios (Table 1).

The results of these experiments reveal two novel observations: (1) they are the first studies to support the hypothesis that pristine SWCNTs have the capability to enhance the infectivity of lung epithelial cells with a strain of influenza A H1N1 virus; (2) that the influence of SWCNTs on viral infectivity does not seem particularly dependent on electronic structure, aggregate size, or stability. These results have significant implications for pathogen susceptibility and they highlight a relatively understudied area of nanomaterial toxicity. Only a few reports to date have probed the ability of nanomaterials to influence pathogen behavior and biological effects. Two studies performed in rodent models showed that sequential exposure of carbon nanotubes and bacteria led to enhanced pulmonary inflammation and fibrosis and decreased pathogen clearance, highlighting the potential impacts of combined exposures to these agents [24,25]. Another study recently reported that pre-exposure to SWCNTs through pharyngeal administration does not modulate the immune response to the parasite *T. gondii* [40] suggesting that the influence of carbon nanotubes on infectious agents may be pathogen specific. Other types of nanomaterials have been shown to possess innate antiviral activity. For example, silver nanoparticles have the ability to inhibit infectivity of HIV-1 by interfering with viral fusion and entry into cells [41]. Carbon nanotubes have also been studied in this capacity and appear to bind HIV-1 in modeled simulations [42]. Greater attention has been given to research devoted to the utility of nanoparticles, including carbon-based materials, for viral detection, vaccine development and drug delivery. However, in most cases, the nanomaterials are specifically engineered for such applications.

### The timing of SWCNT exposure is important in driving increased viral infectivity

**Figure 4:**
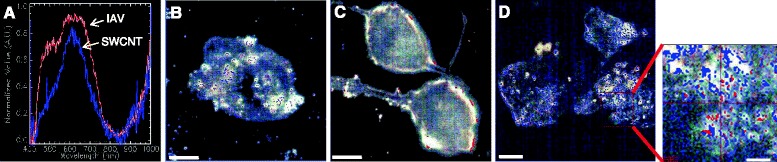
**Mapping of SWCNTs and IAV in SAEC using darkfield hyperspectral imaging microscopy.** Cells were exposed to **(B)** 50 μg/ml SWCNTs, **(C)** 5.0 MOI IAV, or **(D)** SWCNTs + IAV and fixed. **(A)** representative spectral profile for SWCNTs (blue) and IAV (red). Each subsequent panel shows an image of a cell with mapped locations of SWCNTs (blue) and IAV (red) for each condition. For the single exposures, the images show the **(B)** presence of SWCNTs in what appears to be aggregates **(C)** whereas the IAV is present at the cell surface which likely represents budding. **(D)** In the co-treatment the increased localization of the IAV at the cellular edges and within or on the cells is consistent with the increase in titer observed. The SWCNTs also appear to localize in a different pattern compared to the SWCNT treatment only.

In a series of co-exposure experiments we probed whether the pre-treatment period of SWCNTs to SAEC for 24 h was important in influencing viral infectivity. For these studies SAEC were either (1) pretreated with SWCNTs for 2 h, (2) pretreated with virus for 2 h or (3) SWCNTs and virus were incubated together for 3 h *in vitro* prior to exposing the SAEC. In all three conditions, viral infectivity was not impacted compared to virus alone as verified by titer analysis (Table 2). This was not consistent with results from the first experimental condition where cells were pretreated with SWCNTs for 24 h followed by infection with IAV resulting in 5.6 fold increased infectivity (Table 1). The importance of this set of experiments highlights the necessity for an extended pre-treatment period (24 h) of the cells with SWCNTs in order for increased IAV infectivity to occur. These results imply that the SWCNTs altered the cellular environment in some manner to render the cells more susceptible to infection with IAV. The results further indicate that a direct interaction between the SWCNT and IAV may not be a primary mechanism influencing viral infectivity as mixing the SWCNTs and IAV prior to SAEC exposure did not increase infectivity over IAV alone. Carbon nanotubes are quite sorptive and have been shown to interact with proteins, growth factors and other molecules in biological systems [8]. Most studies related to virus-carbon nanomaterial interactions have focused on the utility of such nanoparticles to inhibit viruses. For example, SWCNTs have been postulated to interact with and inhibit an HIV-1 integrase in simulated models [42], whereas another study has shown that carbonaceous materials such as graphene can inhibit viral-mediated cytotoxicity by inducing conformational changes and aggregation of viral proteins through hydrophobic interactions [43]. Using hyperspectral imaging we were able to putatively map the location of SWCNTs and IAV in fixed SAEC preparations to discern whether the particles (tubes and virus) would co-localize, perhaps providing an indication that they were directly interacting. The SWCNTs and IAV were defined by creating unique spectral profiles (Figure 4A). The mapped images show that when cells were exposed only to SWCNTs (Figure 4B) localization of the particles appeared as irregular extracellular aggregates. This pattern was distinct from the localization of IAV (Figure 4C) at the cellular edges and is indicative of classic viral budding and consistent with a 24 h post infection data collection point. Images of the co-exposure condition (Figure 4E) do not show extensive overlapping profiles, suggesting that they may not be interacting physically. However, the localization of the IAV in the co-exposure experiments seems more intracellular compared to the surface localized IAV in the absence of SWCNTs. While we cannot discern the definitive cellular localization of the SWCNTs and IAV from these studies, the results suggest that the presence of SWCNTs may influence IAV behavior. Further studies to confirm the presence of SWCNTs on the cell surface versus internally are warranted in future work.

**Table 2:** **Number of virus particles after 24 h of infection with various treatment scenarios with SWCNTs as determined by titer assay**

**Treatment**	**Titer (24 h post-infection)**	**Fold increase in titer**
Virus only	8.8 × 10^6^TCID_50_/ml	
SG65 24 h then IAV	3.8 × 10^7^TCID_50_/ml	4.3X
SG65 2 h then IAV	8.8 × 10^6^TCID_50_/ml	-(no change)
IAV 2 h then SG65	8.8 × 10^6^TCID_50_/ml	-(no change)
Mix SG65 + IAV then add to cells	8.8 × 10^6^TCID_50_/ml	-(no change)

### Changes in gene and protein expression of immune and viral genes by SWCNTs and pandemic influenza H1N1

**Figure 5:**
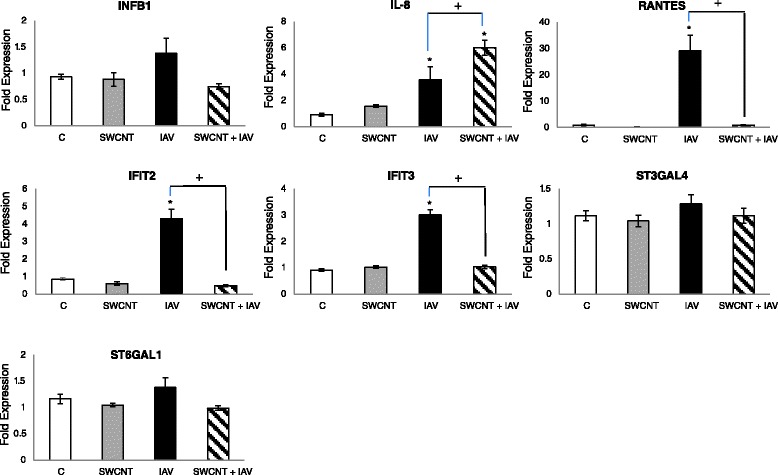
**Changes in inflammatory and anti**-**viral gene expression by SWCNTs and IAV.** SAEC were singly exposed to 50 μg/ml SWCNTs, 5.0 MOI IAV or pre-treated with 50 μg/ml SWCNTs for 24 h followed by exposure to 5.0 MOI IAV for 24 h. qRT-PCR was performed on isolated RNA for the targets shown above. Data are presented as the mean fold expression ± standard error and represent a total of 3 separate experiments. Significant differences in expression levels were determined by ANOVA; *compared to control for each treatment; + significant differences between treatments (*P* < 0.05).

As results from the infectivity studies suggested that the SWCNTs altered the cellular environment such that the cells sense and process the IAV differently, we examined the mRNA expression of a suite of genes relevant to immune and viral responses in lung epithelial cells. For these studies, we performed qRT-PCR measurements for 8 targets (INFβ1, IL-8, RANTES/CCL5, IFIT2, IFIT3, ST3GAL4, ST6GAL1, and IL-10), most of which are typically induced in response to IAV infections both *in vitro* and *in vivo* [5,44-46]. Results from these experiments revealed a number of important findings (Figure 5). First, exposure of SAEC to SWCNTs alone did not significantly modulate the expression of any of the relevant genes. Second, the IAV exposures significantly enhanced the expression of IL-8, RANTES/CCL5, IFIT2 and IFIT3. These results are consistent with prior reports and with a common response of lung epithelial cells to viral exposures [5,46]. Third, the expression of ST3GAL4 and ST6GAL1, sialytransferases that modulate cell surface receptors for influenza virus binding and entry, was not altered by either SWCNTs or IAV. Fourth, IL-10 was not detected in SAEC for any of the treatments and the levels of INFβ1 were low. Finally, the most interesting results are related to the co-exposure experiments with IAV and SWCNTs, which showed a significant reduction in the expression of RANTES, IFIT2 and IFIT3. These targets are early host responses to viruses, serving distinct functional roles in combating infections. RANTES is a potent chemoattractant for monocytes, T lymphocytes, basophils, and eosinophils [47] whereas the IFIT proteins inhibit viral replication by impairing translation through a variety of molecular mechanisms such as destabilizing initiation complexes and interaction with 5’ caps of mRNAs (reviewed by Diamond and Farzan et al., [48]. It is reasonable to speculate that repression of these genes contribute to the impaired ability of the cell to combat viral replication in the presence of SWCNTs This idea is consistent with previous studies using knockdown strategies, which have shown that loss of IFIT expression leads to increased viral infection in a number of cell types [49]. Interestingly, the expression of IL-8, a chemokine also commonly induced by viruses in lung cells and increased by IAV in our SAEC, was not repressed by SWCNTs at the mRNA level but was in fact increased. While the mechanism for this observation is not clear, the data suggest that the signaling mechanisms controlling IL-8 and the IFIT and RANTES genes are not conserved. Complex integrative crosstalk may occur between pathways that control expression of these genes, such as modulation of NF-kB, IRFs and JAK/STAT, which are topics of future study. Further studies that investigate transcriptional profiles over time would also reveal the transient nature of the gene expression. This may account for the surprising lack of induction of INFβ1 and IL-10 by IAV which may have been more responsive earlier in the exposure period. However, it is also possible that INFβ1 and IL-10 are not highly inducible by IAV which may render the SAEC more susceptible to IAV infection. The lack of interferon response is purportedly one reason select cell types support the replication of a variety of virus types [50,51].

**Figure 6:**
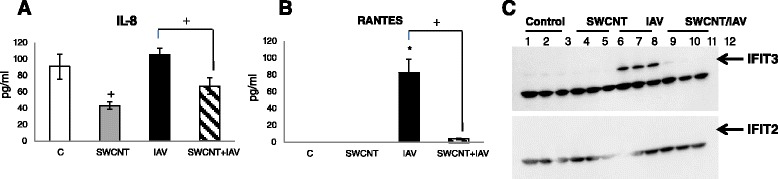
**Changes in expression of IL**-**8**, **RANTES and IFIT proteins.** SAEC were singly exposed to 50 μg/ml SWCNTs, 5.0 MOI IAV or pre-treated with 50 μg/ml SWCNTs for 24 h followed by exposure to 5.0 MOI IAV for 24 h. The levels of **(A)** IL-8 and **(B)** RANTES were quantified by ELISA in cell media and data presented as the mean pg/ml ± standard error for three separate experiments. Significant differences in expression levels were determined by ANOVA; *compared to control for each treatment; + significant differences between treatments (*P* < 0.05) **(C)**. The levels of IFIT3 and IFIT2 were assessed by Western Blot analysis. The expected molecular weight of IFIT3 and IFIT2 is 58 kDa and 55 kDa, respectively. These corresponding areas are marked with an arrow for each blot. The unmarked bands are non-specific targets. The data show the most intense bands for IFIT3 in cells treated with IAV whereas no bands were detectable for IFIT2.

Increased expression of RANTES by IAV and the repression by co-exposure to SWCNTs was quantified at the protein level in cell media at 24 h, which followed the same trend observed for mRNA levels (Figure 6). However, the protein levels of IL-8 showed a significant decrease in SAEC treated with SWCNTs alone compared to controls. Co-treatment of the cells with IAV and SWCNTs also significantly repressed IL-8 levels compared to IAV alone. The distinct and divergent expression profiles produced at the mRNA and protein levels are difficult to interpret without more extensive mechanistic experiments but we speculate that that the timing or perhaps modulation of translational processes contribute to this difference. Using Western Blot analysis we also showed that IFIT3 protein levels were detected only in the IAV treated cells which was consistent with the mRNA profiles. We failed to detect protein levels of IFIT2 despite the induction observed at the mRNA level at the time-point tested.

**Figure 7:**
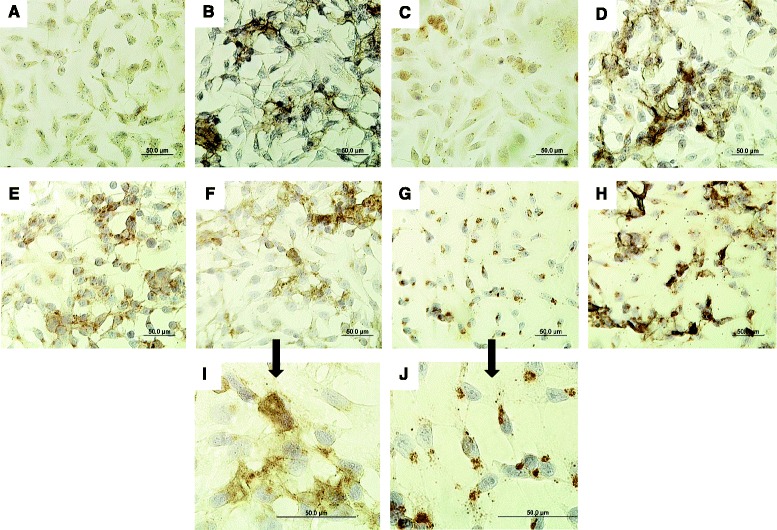
**Detection of sialic acid receptors in SAEC exposed to SWCNTs and IAV.** SAEC were exposed to 50 μg/ml SWCNTs, 5.0 MOI IAV or pre-treated with 50 μg/ml SWCNTs for 24 h followed by exposure to 5.0 MOI IAV for 24 h. Cells were fixed and probed with **(A-D)** SNA lectin or **(E-H)** MAA lectin to detect receptors with α-2,6- to α-2,3-linked sialic acids, respectively. The top panels represent SAEC probed with SNA lectin for the following treatments; **(A)** control, **(B)** SG65 SWCNTs, **(C)** IAV, **(D)** SWCNTs + IAV. The middle panels represent SAEC probed with MAA lectin for the following treatments; **(E)** control, **(F)** SG65 SWCNTs, **(G)** IAV, **(H)** SWCNTs + IAV. The bottom panels are magnified images of SAEC probed with MAA after exposure to **(I)** SWCNTs and **(J)** IAV. Scale bars represent 50 μm. The cell nuclei are stained blue while the receptors are brown in color.

Initiation of viral entry into cells occurs, in part, through a family of cell surface receptors which bind viral hemagglutinin. The primary receptor types for IAV include α2-6-sialylated- and α2-3-sialylated glycans [52]. While we did not see any changes in the mRNA levels for the sialyltransferase enzymes, ST3GAL4 and ST3GAL1, which are responsible for biosynthesizing these receptors so they more readily interact with IAV, detection of the receptors via lectin immunohistochemistry showed more intense staining in SAEC exposed to SWCNTs compared to control and IAV exposed cells (Figure 7). Detection of the α2-6-sialylated receptor by SNA lectin was not abundantly expressed in control of IAV exposed SAEC but seemed to be enhanced in the presence of SWCNTs. Interestingly, positive staining in SWCNT treated cells was not homogeneous but appeared as ‘foci’ and we speculate that these areas correlate to cells in contact with SWCNT aggregates. Increased expression of the α2-6-sialylated receptor would likely allow greater virus particles to enter the cells, assuming this particular virus has a strong interaction with this receptor as supported by the literature [53]. A similar pattern of staining was observed for the α2-3-sialylated receptor detected with lectin MAA. However, basal expression of MAA seemed greater in control cells compared to those probed with SNA. For the IAV treated SAEC the staining appeared more ‘punctate’ for the α2-3-sialylated receptor. It is difficult to speculate as to the precise changes in composition and localization of these receptors and the downstream cellular consequences but these data support their modulation by SWCNTs which support future exploration of sialic acid receptor modulation as a mechanism of SWCNT toxicity.

### SWCNTs alter mitochondrial function in SAEC

**Figure 8:**
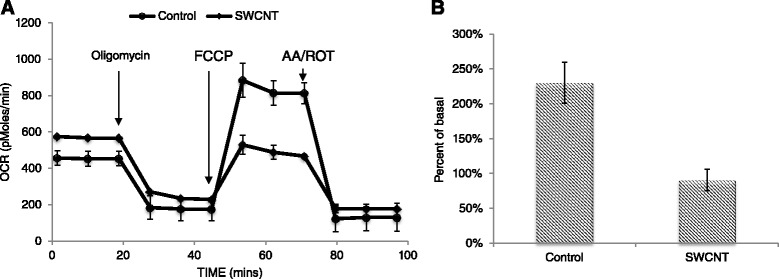
**SAEC mitochondrial respiration during mitochondrial stress test following exposure to SWCNTs.** SAEC exposed to 50 μg/ml SWCNTs or 1% pluronic (control) were subjected to Seahorse Biosciences mitochondrial stress test as described in the methods and oxygen consumption rate (OCR) was measured as the output. **(A)** Data is presented as the mean oxygen consumption rate ± standard error. **(B)** Spare respiratory capacity (% of basal) was calculated by subtracting basal respiration rate from uncoupled oxygen consumption rate. Data is presented as mean spare respiratory capacity ± standard error.

To assess the influence of the nanotubes on another measure of cellular homeostasis we chose to investigate the impact of SWCNTs on mitochondrial function based both on preliminary microarray studies performed by our group with another type of SWCNT (data not shown) and the published body of literature which suggest carbon nanotubes can impact mitochondrial function [54,55]. Using the Seahorse XF24 instrument we measured oxygen consumption rate throughout a mitochondrial stress test in SAEC exposed to SWCNTs (50 μg/ml) for 24 h (Figure 8A) and from this data we calculated differences in spare respiratory capacity (Figure 8B). The most significant observation of these experiments was the ability of SWCNTs to decrease spare respiratory capacity of exposed SAEC compared to control cells. The spare respiratory capacity is commonly used to determine cells ability to cope with stressors (i.e. viruses) that may require increased energy production. Perhaps because cells exposed to SWCNTs were operating at maximal capacity (no spare capacity) they may not be able to adequately respond to viral challenges, resulting in increased infectivity. Since the mitochondria plays a key role in regulating the innate immune response to influenza viruses [56], impairment of mitochondrial function by SWCNTs may limit the typical immune response to viral infections mediated by this organelle.

## Conclusions

It has been well documented that the response of the lung to SWCNTs administered to the respiratory tract can result in robust inflammation, fibrosis, granulomas and pre-cancerous lesions [57-60]. These events are hallmarks of injury induced by other causative particles that lead to fibrosis and cancer, both in humans and animal models. While these studies highlight the potential health impacts of exposure to SWCNTs in isolation, few studies have addressed toxicological impacts associated with combined exposures of nanomaterial and pathogenic agents, with a particular focus on viruses. Results presented here show for the first time that SWCNTs can increase susceptibility of SAEC to IAV infection, possibly by modulating key genes important for viral replication and inflammatory responses, cell receptors that bind IAV and mitochondrial function. Certainly these results warrant translation of these effects to model systems *in vivo*, an emerging focus of our ongoing work. In addition, these studies lay the groundwork for investigating other pathogens and nanomaterials and further elucidating molecular mechanisms of action.

## Materials and methods

### SWCNT and viral preparations

Three types of pristine SWCNTs (SG65, SG76 and CG200) were provided by SouthWest Nanotechnologies Inc. (SWeNT). All SWCNT suspensions were prepared in 1% pluronic F68 (v/v in deionized water, Sigma) using protocols similar to methods previously described [61]. To make suspensions, dry tubes were weighed and added to an appropriate volume of 1% pluronic, sonicated at 30–50 watt power for 25 minutes (Sonifier™ S-450 Digital Ultrasonic Cell Disruptor/Homogenizer, Branson Ultrasonics, Danbury, CT) in an ice bath. SWCNTs were serially diluted in 1% pluronic, their absorbance read at 775 nm (Synergy H1, Biotek). The rest of the stock was centrifuged at 17860 × g for 20 min and the resultant supernatant was sonicated at 30 watt power, diluted, and the absorbance was measured as mentioned above. The concentration of SWCNTs in the supernatant was calculated as follows: [supernatant] = [stock] × [Abs_supernatant_/Abs_stock_]. All working stocks were re-sonicated at 30 watt power for 1 min immediately before using in the experiments. Carbon black particles (Sigma Aldrich) <50 nm in size was employed as a particle control in select studies.

Influenza virus H1N1 strain A/Mexico/4108/2009 was kindly provided by Drs. Gary Heil and Gregory Gray at the University of Florida and was propagated in MDCK cells in serum-free medium with 1 μg/ml L-1-tosylamide-2-phenylethyl chloromethyl ketone (TPCK)-treated trypsin.

### Dynamic light scattering, static light scattering and metal analysis of SWCNT

Dynamic light scattering (DLS) analysis using a goniometer system (ALV-CGS/3, ALV-GmbH, Langen, Germany) was employed to evaluate stability of SWCNTs suspended in pluronic F68 solution over a 24 h period. A detailed measurement procedure has been described elsewhere [4,33]. In brief, a SWCNT sample was introduced to the sample chamber in a cleaned borosilicate vial (Fisher Scientific, Pittsburg, PA). A 632.8 nm laser was shined through the sample and scattered light was collected at an angle of 90° in 15 s intervals for 1 h. Hydrodynamic radii (HR) of the nanoparticles were calculated by the software interface using the collected time-dependent scattering data. HR of the particles were then plotted against measurement time to see the stability in this time period. This procedure was repeated in every 10 s for 24 h to monitor aggregate size change.

The ALV-CGS/3 goniometer system (ALV-GmbH, Langen, Germany) was also utilized to perform angle-dependent static light scattering (SLS) of pluronic suspended SWCNTs. For these measurements SWCNTs were added to a borosilicate glass vial and angle-dependent scattering was performed for an angular range of 12.5° to 100° with 0.5° increment. Fractal dimension was computed from log-log profiles of scattering intensity and wave vector. Slope of the fractal regime profile was then computed using linear fit which represented the fractal dimension [32]. Data was collected initially and after 2, 6, 12 and 24 h upon exposure of the SWCNTs to the media. Analysis of trace metal composition within SWCNTs and in cell culture media exposed to SWCNT leachate was performed by inductively coupled plasma-mass spectrometry (ICP-MS) using methods previously described [61]. Analysis was performed on triplicate SWCNT samples (20 mg dry weight) and in cell media after incubation with SWCNTs for 48 h. See supplemental data section for complete results of analysis (Additional file 1: Table S1).

### Cell viability assay

Human small airway epithelial cells (SAEC) were obtained from Dr. Brooke Mossman and previously characterized by Hei and colleagues [62]. Cells were seeded in 24-well plates in RPMI 1640 advanced medium supplemented with 10% FBS. Stock solutions of SWCNTs were added at final concentrations of 12.5 μg/ml, 25 μg/ml, 50 μg/ml and 100 μg/ml. After 24 h, cell viability was assessed using a standard trypan blue dye exclusion technique where the number of live and dead (blue) cells were counted under a light microscope with a hemocytometer. A total of 200 cells were counted per treatment (n = 3) and the entire experiment was repeated 3 times. Data are graphed as the mean and standard error of all experiments combined.

### SWCNT and virus co-exposures to lung cells

SAEC were seeded onto cover-slips in 6-well plates in complete RPMI media and grown overnight. When the cells reached ~90% confluency, they were washed and 2 ml of serum free media were added. Appropriate volume of SG65 SWCNTs was added for a final concentration of 50 μg/mL. In control wells, equal volume of pluronic F68 was added. After 24 h, two different doses of virus (at MOI of 0.1 and 0.5) were added in designated wells. After 24 h of exposure the media was collected and stored for viral titer assays. The cells were fixed with cold 80% acetone and 20% methanol for immunofluorescence staining (see below). Similar exposures were performed where cells were pretreated with 50 μg/mL SG76 and CG200 SWCNTs followed by viral infections (high dose only).

Three additional treatment regimens were performed with SG65 SWCNTs; cells were pretreated with the SWCNTs for 2 h followed by infection with the high dose of virus; cells were first infected with virus for 2 h followed by treatment with 50 μg/mL SG65 SWCNTs; SG65 SWCNTs and virus were mixed together and incubated at 33°C for 3 h on a rotating platform and then added to the cells. Viral titers were performed after 24 h. Duplicates were assayed for each treatment. The entire experiment was repeated 3 times and data presented as the mean titer value for the experiments combined.

### Immunofluorescence staining of cells for virus

For detection of IAV, the fixed cells were first stained for nucleoprotein A (Anti-Influenza A nucleoprotein, Clone A1, A3 Blend, Millipore Cat# MAB8251) by incubating the cells with antibody (1:100 diluted in PBS) for 1 h at 37°C. Next, the cells were washed three times with PBS and then incubated with the fluorescent labeled secondary antibody (Anti-Mouse IgG, FITC conjugated, Millipore Cat # AQ303F, 1:100 diluted in PBS) for 1 h at 37°C. Finally, the cells were washed three times in PBS and the cover slips were mounted onto microscope slides for observation under a fluorescence microscope. Triplicates were assayed for each treatment where 200 cells were counted per slide (n = 2) and the experiment was repeated 3 times. Data are presented as the mean and standard error for each treatment for the 3 experiments combined.

### Viral titers (endpoint dilution assay)

To quantify the number of virus particles we calculated TCID_50_ values which is the dilution of virus required to infect 50% of inoculated cells. To perform these assays, MDCK cells were seeded in 96 well plates. Serial dilutions (10^−1^, 10^−2^, 10^−3^, 10^−4^, 10^−5^, 10^−6^) of virus stock and supernatant from exposed SAEC as described above were added to MDCK monolayers. Cells were incubated at 33°C and monitored for cytopathic effects by light microscopy (CPE) at 5 days post infection. Each well that displayed CPE were scored as positive. The TCID_50_ is calculated to be the dilution of virus at which 50% of the cell cultures are infected. Duplicates were assayed for each treatment. The entire experiment was repeated 5 times (Table 2) or 3 times (Table 1) and data presented as the mean titer value for the experiments combined.

### Enhanced darkfield hyperspectral imaging microscopy

An optical microscope (Olympus BX41) equipped with a CytoViva optical condenser and a hyperspectral imaging (HSI) spectrometer (CytoViva Hyperspectral Imaging System, Auburn, AL) was used to collect darkfield and hyperspectral images of SAEC treated with viruses and/or SWCNTs. HSI analysis was performed using a previously described procedure [63,64]. Using a 100× objective the samples were scanned (512 lines with a step size of 10 nm) in the visible and near-infrared wavelengths (VNIR: 400–1000 nm) with a bandwidth of 1.5 nm. The spatial and spectral information was derived from each image using the Environment for Visualization (ENVI) v4.8 software. For each sample, three to four images were collected at a constant high gain with 2 s exposure time and background subtracted using 10 dark-current images obtained prior to the sample scan. Using ENVI Spectral Hourglass Wizard the endmembers (spectrally dominant pixels) of IAV and SWCNTs were derived from hyperspectral images of SAEC co-exposed with viruses or SWNTs (controls). These endmembers were used for mapping. Spectral analysis of endmembers from controls (IAV or SWNTs) and the experiment (IAV + SWCNTs) confirmed the presence of spectral fingerprints of viruses and SWCNTs in the experiment. The hyperspectral classification images were generated using spectral angle mapper method with threshold set at 0.25. Analysis was done on one experiment with triplicate coverslips per treatment. A total of 10 images per slide was analyzed.

### Expression levels of immune and viral-related genes in lung cells

Changes in mRNA expression in SWCNT and virus exposed lung cells as described above was performed by qRT-PCR using methods previously described [65]. Briefly, RNA was isolated from cells using the RNeasy Mini Kit (Qiagen), quantified and reverse transcribed (Promega). The resultant cDNA was amplified using validated primers and probes specific to each gene target; RANTES, IL-8, IFIT2. IFIT3, RANTES, INFβ1, ST6GAL1, ST3GAL4, IL-10 (Applied Biosystems). Expression of GAPDH was employed for a standard housekeeping gene and all data is presented as normalized fold change in expression compared to controls using the delta delta Ct method. Since no IL-10 was detected in SAEC, to validate the IL-10 primers RNA from Namalwa cells was tested as a positive control. Namalwa cells are B lymphocytes that are immortalized by Epstein-Barr virus (EBV) and express IL-10. For this experiment the cells were cultured in advanced RPMI 1640 medium with 2 g/L D-Glucose, 1.0 mM Sodium Pyruvate, 2 g/L Sodium Bicarbonate, 1% GlutaMax (Invitrogen), 1% PSN (Invitrogen), and 7.5% fetal bovine serum (Thermo Fisher Scientific Inc.). Cells were collected and their RNA was extracted for qRT-PCR as described above. For all genes, triplicate samples were assayed for each treatment. The entire experiment was repeated 3 times and data presented as the mean and standard error for the combined experiments.

### Secretion of IL-8 and RANTES in SAEC media

Levels of IL-8 and RANTES in cell culture supernatants were measured using a human CXCL8/IL-8 Quantikine ELISA kit (R&D Systems, MN) and human CCL5/RANTES Quantikine ELISA Kit (R&D Systems), respectively, following the manufacturer’s instructions. Absorbance was measured using a Synergy H1 hybrid multi-mode microplate reader with Gen 5 software (BioTek, VT). Triplicates were assayed for each treatment. The entire experiment was repeated 3 times and data presented as the mean and standard error for the experiments combined.

### Detection of IFIT proteins by western blot analysis

Cells were exposed to SWCNTs and IAV as describe above, the media removed and cells washed in PBS. The cells were then harvested in Pierce RIPA buffer (Thermo Fisher Scientific Inc., MA). The total protein concentration was quantified and equal amounts of protein for each sample (80 μg) was separated on a 10% polyacrylamide gel and transferred to nitrocellulose membrane. The blots were incubated with IFIT2 (F-12, 1:200 dilution, Santa Cruz Biotechnology, Inc., TX), IFIT3 (B-7, 1:200 dilution, Santa Cruz Biotechnology, Inc) or β-actin (AC-15, 1:5000 dilution, Sigma-Aldrich, MO) overnight at 4°C. After three washes with TBST, the blots were incubated with rabbit anti-Mouse IgG (H + L) HRP-conjugated secondary antibody (1:2500 dilution, Thermo Fisher Scientific Inc., MA) for 2 h at room temperature. Following three washes with TBST, standard detection was carried out using Novex® ECL chemiluminescent substrate reagent kit (Invitrogen, NY) according to manufacturer’s instruction. Images were scanned using a ChemiDoc™ MP Imaging System with Precision Melt Analysis™ software 1.2 (Bio-Rad, CA).

### Lectin immunohistochemistry

SAEC plated on coverslips were fixed in acetone, washed with PBS, and endogenous peroxidase was quenched by incubation BLOXALL™ Blocking Solution (Vector Laboratories, CA) for 10 min at room temperature. Cells were then washed three times with PBS and blocked in 3% (w/v) bovine serum albumin (BSA) for 1 h at 4°C prior to adding 20 μg/ml biotinylated Maackia amurensis lectin II (MAA, Vector Laboratories) or 10 μg/ml biotinylated Sambucus nigra (elderberry) bark lectin (SNA, Vector Laboratories) overnight at 4°C. After three washes with PBS, the slides were then incubated with 5 μg/ml horseradish peroxidase streptavidin (HRP, Vector Laboratories) for 1 h at room temperature. After another three washes with PBS, Vectastain® ABC kit (Cat # PK-4000, Vector Laboratories) was used to detect the biotinylated lectins following the manufacturer’s instructions. The cells were counterstained with Hematoxylin QS (Vector Laboratories) for 10 s, washed, and mounted with VectaMount® permanent mounting solution (Vector Laboratories). Images were taken using a Nikon ECLIPSE Ti-U microscope (Nikon Instruments Inc., NY) with QImaging camera (Q-Capture Pro 7.0.5 software installed, BC, Canada). Analysis was done on two experiments with duplicate coverslips per treatment. A total of 10 images per slide was captured and analyzed.

### Assessment of mitochondrial function

Measurements of basal respiration rate and mitochondrial stress were performed on a seahorse XF24. For these experiments, SAEC were plated at 20,000 cells/well in XF 24 microplates, allowed to adhere overnight, and exposed to SG65 SWCNTs or 1% pluronic (control). After 24 h following exposure, media was removed, rinsed twice, and replaced with XF mito stress test media (DMEM, 2 mM L-glutamine, 2 mM sodium pyruvate, 10 mM glucose). Basal respiration rates were measured three times, followed by injection of oligomycin (1 μM final concentration). Oligomycin inhibits ATP synthase allowing for measurement of mitochondrial respiration associated with ATP production. Three respiration rates were again measured followed by injection of Carbonyl cyanide-4(trifluoromethoxy)phenylhydrazone (FCCP, 0.5 μM final concentration). FCCP collapses the mitochondrial proton gradient causing uninhibited electron flow, resulting in maximal oxygen consumption. Maximal respiration rates were measured three times followed by an injection of rotenone (ROT, 1 μM final concentration) and antimycin A (AA, 1 μM final concentration). These two inhibitors completely shut down mitochondrial respiration, allowing for measurement of non-mitochondrial respiration in the cell. The spare respiratory capacity was calculated by subtraction of basal respiratory rate from maximal respiratory rate

### Statistical analysis

SigmaPlot version 12.0 (Systat Software Inc., San Jose, CA) software for Windows was used for all statistical analysis. ANOVA with Tukeys was used to analyze differences between treatments. Null hypothesis was rejected at a p-value < 0.05.

## Additional file

Additional file 1: Table S1. Concentration of metals (in ppb) in media after 24 h of incubation of SWCNTs with RPMI media.
