# Supplementary material for: Single-walled carbon nanotubes increase pandemic influenza A H1N1 virus infectivity of lung epithelial cells
Source: Part Fibre Toxicol. 2014 Dec 14;11:66. doi: 10.1186/s12989-014-0066-0 (PMC4318452; doi:10.1186/s12989-014-0066-0)
Supplement: Additional file 1: Table S1. — Concentration of metals (in ppb) in media after 24 h of incubation of SWCNTs with RPMI media. [file 12989_2014_66_MOESM1_ESM.pdf]

Table S1. Concentration of metals (in ppb) in media after 24 h of incubation of SWCNTs with RPMI media

**Metal Ion**	**RPMI media**	**SG65**	**SG76**	**CG200**
Cu	ND	ND	ND	ND
Zn	1405.943	1244.323	1980.234	2113.427
Cr	ND	ND	ND	ND
Fe	114.782	73.140	179.595	98.561
Co	ND	5.430	ND	1.826
Ni	ND	ND	ND	ND
Sr	8.799	8.473	9.054	8.869
Mo	ND	23.819	125.621	117.284
Ag	0.090	0.025	0.077	0.114
Al	273.589	238.969	421.321	393.811
Na	3111995.646	3073128.649	2951064.539	2953590.844
Mg	9696.327	9484.667	9073.010	9047.634
Ca	20349.665	19238.210	20136.259	20494.612
K	199072.054	196172.688	188775.004	190400.743
Be	ND	ND	ND	ND
As	ND	ND	ND	ND
Cd	ND	ND	ND	ND
Ba	102.476	89.360	97.837	98.182
Ce	0.175	0.213	0.631	0.228
Ti	ND	ND	ND	ND
Pb	ND	ND	ND	ND
Th	ND	ND	ND	ND
U	ND	ND	ND	ND
V	ND	ND	ND	ND

ND = non detect
